# Interaction of RECQL4 with poly(ADP‐ribose) is critical for the DNA double‐strand break response in human cells

**DOI:** 10.1002/2211-5463.13917

**Published:** 2024-10-27

**Authors:** Sunyoung Shin, Dongmin Kim, Hyemi Kim, Won‐Ho Cho, Gyungmin Kim, Joon‐Kyu Lee

**Affiliations:** ^1^ Department of Biology Education Seoul National University Korea; ^2^ Interdisciplinary Graduate Program in Genetic Engineering Seoul National University Korea

**Keywords:** DNA double‐strand breaks, DNA repair, poly(ADP‐ribose) binding motif, poly(ADP‐ribosyl)ation, RECQL4

## Abstract

To overcome genotoxicity, cells have evolved powerful and effective mechanisms to detect and respond to DNA lesions. RecQ Like Helicase‐4 (RECQL4) plays a vital role in DNA damage responses. RECQL4 is recruited to DNA double‐strand break (DSB) sites in a poly(ADP‐ribosyl)ation (PARylation)‐dependent manner, but the mechanism and significance of this process remain unclear. Here, we showed that the domain of RECQL4 recruited to DSBs in a PARylation‐dependent manner directly interacts with poly(ADP‐ribose) (PAR) and contains a PAR‐binding motif (PBM). By replacing this PBM with a PBM of hnRNPA2 or its mutated form, we demonstrated that the PBM in RECQL4 is required for PARylation‐dependent recruitment and the roles of RECQL4 in the DSB response. These results suggest that the direct interaction of RECQL4 with PAR is critical for proper cellular response to DSBs and provide insights to understand PARylation‐dependent control of the DSB response and cancer therapeutics using PARylation inhibitors.

## Graphical abstract

Here, we showed that RECQL4 protein contains a poly(ADP‐ribose)‐binding motif (PBM). The interaction of RECQL4 with poly(ADP‐ribose) via its PBM is critical for recruitment to DNA double‐strand break (DSB) sites and proper cellular response to DSBs. These results provide insights for understanding the poly(ADP‐ribosyl)ation‐dependent control of the DSB response and cancer therapeutics using poly(ADP‐ribosyl)ation inhibitors.

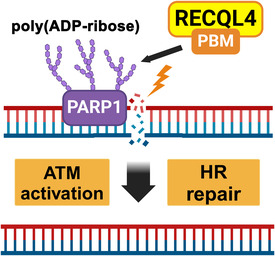

**Abbreviations**
ATM: ataxia telangiectasia mutated
DDRs: DNA damage responses
DSBs: DNA double‐strand breaks
HR: homologous recombination
MRN: MRE11‐RAD50‐NBS1
NCS: neocazinostatin
NLS: nuclear localization signal
PAR: poly(ADP‐ribose)
PARG: poly(ADP‐ribose) glycohydrolase
PARP: poly(ADP‐ribose) polymerase
PARylation: poly(ADP‐ribosyl)ation
pATM: phosphor‐ATM
PBM: PAR‐binding motif
SSBs: single‐strand breaks

The human genome is continuously threatened by a variety of genotoxic stresses that cause DNA damage [1]. To overcome these stresses and maintain genome integrity, cells have evolved powerful and effective DNA damage responses (DDRs), which detect and respond to DNA lesions [2]. RECQL4 plays an important role in the DDR, especially in the cellular response to DNA double‐strand breaks (DSBs), the most cytotoxic type of DNA damage [3, 4, 5].

RECQL4 is a member of the RecQ helicase family that is conserved in multiple species and has been implicated in the maintenance of genome integrity [6]. RECQ family helicases, including RECQL1, WRN, BLM, RECQL4, and RECQL5, display extensive homology in their helicase domains and reportedly, play key roles in the DDR [7, 8]. Furthermore, mutations in BLM, WRN, and RECQL4 are associated with Bloom, Werner, and Rothmund‐Thomson syndromes, respectively, all of which present genome instability and cancer predisposition [9]. RECQL4 has been shown to be rapidly recruited to DSB sites [10], and its helicase activity is crucial for both homologous recombination (HR) repair [3, 11] and activation of a checkpoint kinase, Ataxia telangiectasia mutated (ATM), which plays an essential role in the DSB response [4]. RECQL4 physically interacts with the MRE11‐RAD50‐NBS1 (MRN) complex and CtIP and has been postulated to participate in DNA 5′‐end resection, which is an indispensable stage of HR repair [3]. However, a recent study has shown that 5′‐end resection occurs even in the absence of RECQL4 as long as the MRN complex remains stable [11]. Moreover, RECQL4 plays a critical role in stabilizing the MRN complex during the DSB response through its interaction with the deubiquitinase USP2 [12]. Therefore, RECQL4 appears to carry out its role in HR repair by regulating the stability of the MRN complex rather than directly participating in the repair process.

The RECQL4 protein was shown to be rapidly recruited to DSB sites, and its recruitment is prevented by inhibition of poly(ADP‐ribosyl)ation (PARylation), a post‐translational modification that plays an important role in the DDR [13]. However, the mechanism of PARylation‐dependent recruitment, as well as its significance in the DSB response, have not yet been defined. In this study, we determined the region of RECQL4 required for PARylation‐dependent recruitment to DSB sites and investigated its interaction with poly(ADP‐ribose) (PAR). Further, we aimed to clarify the significance of REQL4‐PAR interaction in human cells by analyzing the influence of PAR‐binding motif (PBM) replacement on the role of RECQL4 in the DSB response. The results of this study are anticipated to enhance our understanding of RECQL4 function and shed light on the implications of PARylation‐dependent control of the DSB response in human cells.

## Materials and methods

### Cell culture and reagents

Human osteosarcoma (U2OS) cells were cultured in Dulbecco's modified Eagle's medium (Welgene, Gyeongsan, Korea) supplemented with 10% fetal bovine serum (Welgene) and 1% antibiotic‐antimycotic solution (Welgene). PARP‐KO‐U2OS cells were obtained from S. Zha at Columbia University and cultured in Dulbecco's modified Eagle's medium (Welgene) supplemented with 15% fetal bovine serum (Welgene) and 1% antibiotic‐antimycotic solution (Welgene). These cells were maintained in a humidified atmosphere of 5% CO_2_ at 37 °C. The transfection of plasmids was performed with Lipofectamine 3000 (Invitrogen, Waltham, MA, USA). For the depletion of proteins, siRNAs were transfected using a Neon electroporator (Invitrogen) and incubated for 48 h. All siRNAs utilized in this study were custom‐synthesized by Bioneer (Daejeon, Korea). The sequences of the sense strand of siRNA duplexes are as follows: GL (targeting firefly luciferase), 5′‐AACGUACGCGGAAUACUUCGA‐3′; RECQL4, 5′‐GACUGAGGACCUGGGCAAA‐3′. To induce DNA DSBs, the cells were treated with neocazinostatin (NCS) (N9162; Sigma‐Aldrich, Burlington, MA, USA) or bleomycin (M2100; AbMole, Houston, TX, USA). The PAR polymerase (PARP) inhibitor, olaparib, was purchased from Selleckchem. The PAR glycohydrolase (PARG) inhibitor, PDD00017273, and ATM inhibitor, KU‐55933, were purchased from Sigma‐Aldrich. The DNA‐dependent protein kinase (DNA‐PK) inhibitor, NU7441 was obtained from Axon Medchem, Groningen, Netherlands. Protease inhibitor cocktail was purchased from GenDEPOT (P3100‐001), Katy, TX, USA. Expression vectors for mutant RECQL4 proteins (Walker A, Walker B, and helicase domain deletion) were generated in a previous study [4]. For truncated RECQL4 protein expression, various regions of RECQL4 cDNA (amino acids 1–247 for CD1, 1–492 for CD2, 1–835 for CD3, 820–1208 for ND1, 437–1208 for ND2, 241–1208 for ND3, 241–437, and 360–437) were amplified by PCR and inserted between the BamHI and HindIII sites in pcDNA3.1(−) plasmids containing enhanced green fluorescent protein (EGFP) and the nuclear localization signal sequence (NLS) of Simian virus 40.

### Laser microirradiation and real‐time imaging of fluorescent proteins

The cells grown on a dish with a thin glass bottom were treated with 5 μg*·*mL^−1^ Hoechst 33342 (ThermoFisher Scientific, Waltham, MA, USA) 10 min before microirradiation. Cells were locally irradiated with a fixed laser wavelength (405 nm) at a scan speed of 32.77 μs·pixel^−1^ with four iterations using an LSM880 laser confocal microscope system with a temperature‐controlled CO_2_ chamber (Zeiss, Oberkochen, Land Baden‐Württemberg, Germany). The Plan‐Apochromat 63× oil objective lens was used to observe the time‐lapse images, and the fluorescence intensities of irradiated areas relative to those of non‐irradiated areas within the nucleus were measured using the ZEISS zen 2.3 SP1 software (Zeiss).

### Immunoprecipitation and immunoblotting

For immunoprecipitation, the cells were lysed in a buffer A (40 mm Tris/HCl, pH 7.5, 100 mm NaCl, 2.5 mm MgCl_2_, 1 mm DTT, 5% glycerol, and protease inhibitor cocktail) with 0.2% NP‐40. After sonication, the cell lysate was cleared by centrifugation at 18 000 ***g*** for 10 min, and the supernatant was used for immunoprecipitation. The extracts were incubated for 2 h with specific antibodies, and then further incubated for 1 h with Protein A‐agarose beads (P9201‐100; GenDEPOT). Beads were pelleted by centrifugation and washed for three times with a buffer A with 0.05% NP‐40. To prepare whole‐cell extracts for immunoblotting, the cells were lysed in a buffer containing 40 mm Tris/HCl (pH 7.5), 150 mm NaCl, 1% NP‐40, 1 mm ethylenediaminetetraacetic acid (EDTA), 0.25% sodium deoxycholate, 20 mm NaF, 0.1 mm sodium orthovanadate, and protease inhibitors. The cells were sheared by sonication, and the concentration of proteins was measured using Bradford assay. The following antibodies were used for immunoprecipitation and immunoblotting: anti‐RECQL4 antibody, which was prepared as previously described [4], anti‐PAR (4335‐MC‐100; Bio‐Techne, Minneapolis, MN, USA), anti‐Lamin B1 (ab16048; Abcam, Cambridge, England, UK), anti‐GFP (SC‐9996; Santa Cruz, Dallas, TX, USA), anti‐FLAG (AC063; AB Frontier, Seoul, Korea), anti‐HA (AE008; ABclonal, Woburn, MA, USA), anti‐mouse IgG (GTX213111‐01; GeneTex, Irvine, CA, USA), and anti‐rabbit IgG (GTX213110‐01; GeneTex).

### Immunofluorescence staining

For immunostaining of MRE11, RAD51, and phospho‐ATM (pATM), U2OS cells grown on coverslips were pretreated with a cold buffer containing non‐ionic detergent (10 mm Pipes pH 7.0, 100 mm NaCl, 300 mm sucrose, 3 mm MgCl_2_, and 0.5% Triton X‐100) on ice for 5 min and fixed with 4% paraformaldehyde in PBS for 10 min at 25 °C. The fixed cells were washed with PBS containing 0.25% Triton X‐100 on ice for 10 min and then incubated in a blocking buffer (5% bovine serum albumin and 0.25% Triton X‐100 in PBS) for 30 min at 25 °C. The indicated proteins were labeled consecutively with the respective primary and secondary antibodies in the blocking buffer for 1 h at 25 °C. The nuclei of the cells were stained with 0.1 μg·mL^−1^ 4′‐6′‐diamidino‐2‐phenylindole (DAPI) in mounting solution (VECTASHIELD, H‐1200; Vector, Burlingame, CA, USA) in the dark. Fluorescent images were obtained by fluorescence microscopy (LSM880; Zeiss), and cells containing 20 or more foci were counted as foci‐positive cells for quantitation. Anti‐pATM (S1981; Cell Signaling, Danvers, MA, USA), anti‐MRE11 (GTX30294; GeneTex), anti‐RAD51 (N1C2; GeneTex), mouse anti‐γH2AX (Sc‐517348; Santa Cruz), and rabbit anti‐γH2AX (A300‐081A‐M; BETHYL, Montgomery, TX, USA) antibodies were used in this study.

### Preparation of GST‐RECQL4^360–437^ protein and PAR binding assay

For the preparation of GST‐RECQL4^360–437^ proteins, the cDNA fragment encoding the amino acids 340–437 region of RECQL4 was amplified using PCR and cloned into the pGEX‐5X‐1 plasmid. For expression of GST and GST‐RECQL4^360–437^ proteins, pGEX‐5X‐1 and recombinant plasmids were transformed to BL21‐CodonPlus(DE3)‐RIPL cells. BL21‐CodonPlus(DE3)‐RIPL cells harboring plasmids were cultured at 37 °C in 200 mL LB medium containing 100 μg·mL^−1^ ampicillin, and proteins were induced (at an OD_600_ 0.4) with 1 mm isopropyl‐1‐thio‐β‐d‐galactopyranoside for 4 h at 37 °C. Cells were harvested and resuspended in 10 mL of buffer B (50 mm Tris/HC1, pH 8.0, 150 mm NaC1, 1 mm DTT, and protease inhibitors) containing 1% Triton X‐100. Cells were incubated for 1 h on ice in buffer B with 50 μg·mL^−1^ lysozyme, disrupted by sonication, and centrifuged at 18 000 ***g*** for 30 min at 4 °C. The supernatant was mixed with 100 μL of glutathione Sepharose 4B beads, and incubated at 4 °C for 2 h. The beads were washed three times with buffer B containing 0.1% Triton X‐100. Then, bound proteins were eluted with buffer B containing 20 mm reduced glutathione. After removal of glutathione using the Sephadex G25 (GE HealthCare, Chicago, IL, USA) desalting column, about 100 μg of GST and 37 μg of GST‐RECQL4^360–437^ proteins were obtained and used for the PAR‐binding assay.

For the GST pull‐down assay, 20 pmol of biotin‐labeled PAR polymer (4336–100‐02; Trivigen, Gaithersburg, MD, USA) was mixed with the indicated amounts of GST‐RECQL4^360–437^ or GST proteins in a binding buffer (30 μL) containing 20 mm Tris/HCl (pH 8.0), 100 mm NaCl, 1 mm MgCl_2_, and 0.1 mm EDTA. After incubation at 25 °C for 20 min, reaction mixtures were further incubated with glutathione Sepharose 4B beads at 4 °C for 30 min. Beads were washed three times with 0.5 mL of binding buffer, and PAR polymers bound to beads were recovered by boiling at 90 °C for 8 min in the elution buffer (20 μL) containing 50 mm Tris/HCl (pH 6.8), 2% SDS, and 1% β‐mercaptoethanol. Eluted samples were loaded onto a nylon membrane using a dot‐blot apparatus (Cleaver Scientific, Rugby, Warwickshire, UK). After crosslinking by UV‐irradiation and drying at 60 °C for 20 min, immunoblotting was carried out using an anti‐PAR antibody.

For the PAR pull‐down assay, 30 pmol of biotin‐labeled PAR polymer (4336‐100‐02; Trivigen) was mixed with 30 pmol of GST or GST‐RECQL4^360–437^ proteins and streptavidin beads in a binding buffer (60 μL) containing 20 mm Tris/HCl (pH 8.0), 100 mm NaCl, 1 mm MgCl_2_, 0.1 mm EDTA, and 0.1 mg·mL^−1^ bovine serum albumin. After incubation at 4 °C for 2 h, beads were washed three times with the binding buffer, and proteins remaining on the beads were subjected to SDS/polyacrylamide gel electrophoresis for immunoblotting with an anti‐GST antibody.

### WST‐1 assay for cell viability

The assay was carried out as described in the manufacturer's instruction (EZ‐Cytox; DoGenBio, Seoul, Korea). In brief, U2OS cells transfected with the indicated siRNAs and expression plasmids were cultured in 96‐well plates for 48 h. Cells were exposed to different concentrations of bleomycin. After incubation for 24 h, the cells were treated with 10% WST‐1 reagent for 1 h, and absorbance was measured using an Epoch2 Microplate Spectrophotometer (BioTek, Winooski, VT, USA).

### Statistical analysis

Statistical significance between groups was determined by two‐tailed Student's *t* test using graphpad prism 8 software (Graphpad, La Jolla, CA, USA). Data are presented as mean ± SD or SEM. All of the statistical tests performed are indicated in the figure legends.

## Results

### The recruitment of RECQL4 to DNA DSB sites requires the activity of PARP

**Fig. 1:**
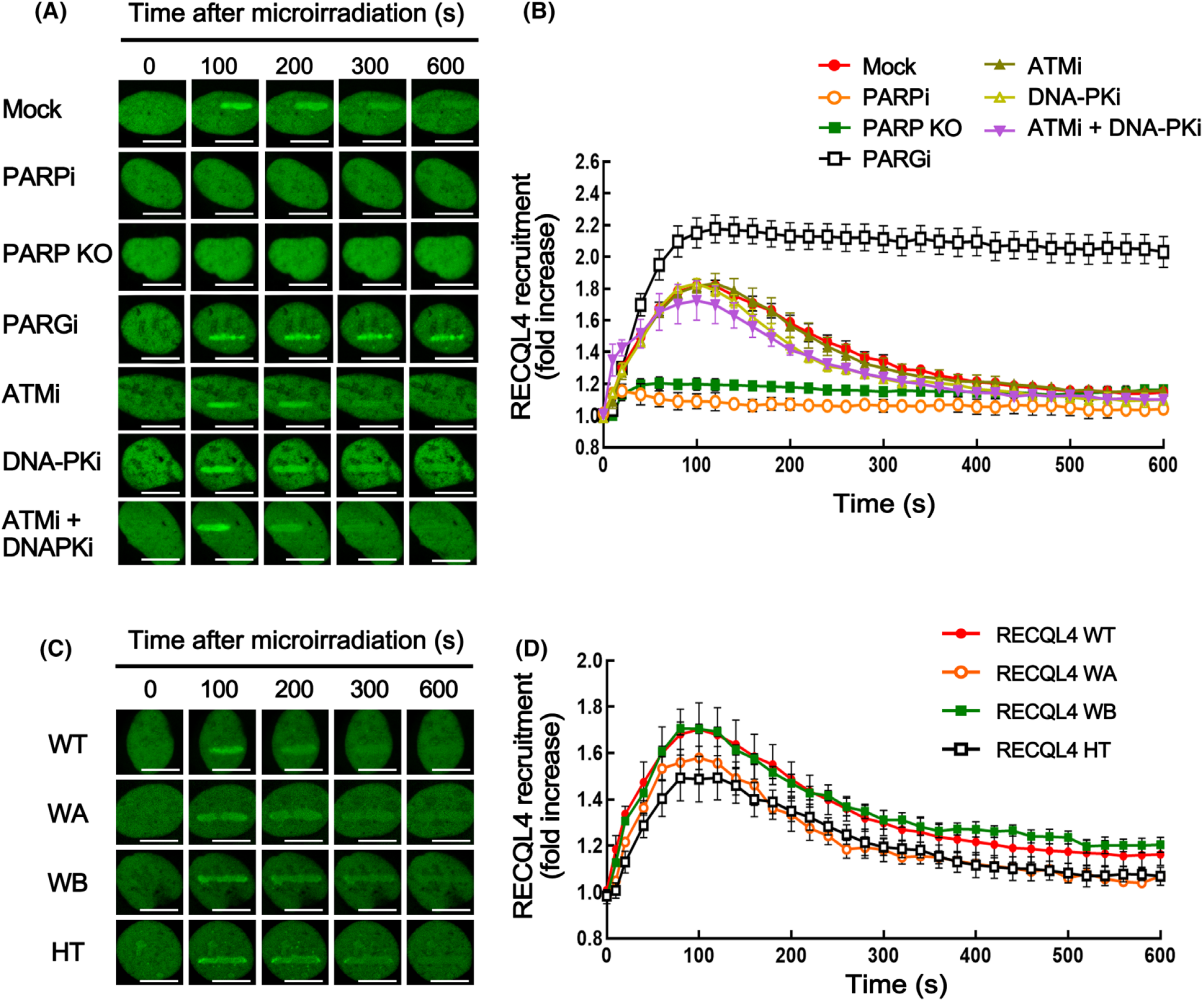
The recruitment of RECQL4 to DSB sites requires the activity of PARP‐1. (A, B) U2OS or PARP1‐KO‐U2OS cells were transfected with EGFP‐RECQL4. EGFP‐RECQL4 transfected U2OS cells were mock‐treated (DMSO) or treated with an inhibitor of PARP (PARPi, 10 μm olaparib), ATM (ATMi, 10 μm KU‐55933), DNA‐PK (DNA‐PKi, 10 μm NU7441), or PARG (PARGi, 1 μm PDD00017273) for 1 h, and EGFP‐RECQL4 binding to microirradiation sites was determined. Representative images (A) and graphs (B) are shown. (C, D) EGFP‐tagged RECQL4 derivatives were expressed in U2OS cells and their binding to laser microirradiation sites was determined. Representative images (C) and graphs (D) are shown. HT, helicase truncated RECQL4; WA, Walker A mutant; WB, Walker B mutant; WT, wild‐type. The scale bar represents 10 μm. Data in graphs are means ± SEM; *n* = 15 from three independent experiments.

To investigate the mechanism of RECQL4 recruitment to DSB sites, we first examined the binding of EGFP‐fused RECQL4 (EGFP‐RECQL4) proteins to laser‐induced DSB sites in the presence of various inhibitors that influence the sensing and signaling of DSBs. As shown in Fig. 1A,B, rapid and transient recruitment of EGFP‐RECQL4 to the microirradiation site was almost completely abolished by treatment with the PARP inhibitor, which prevented PARylation, while dissociation of EGFP‐RECQL4 from DSB sites was prevented by inhibition of PARG, degrading PAR. Furthermore, rapid recruitment of EGFP‐RECQL4 to the microirradiation site was not observed in PARP1 knockout cells. On the other hand, inhibition of ATM and DNA‐PK, important regulators of DSB signaling, did not influence the interaction of RECQL4 with DSBs (Fig. 1A,B). As the helicase activity of RECQL4 was shown to be required for its role in the DSB response [3, 4, 11], we also tested whether the helicase activity of RECQL4 is required for its recruitment to DSB sites. All three mutant proteins defective in their helicase activity retained the ability to bind to and dissociate from DSBs, similar to that of wild‐type RECQL4 (Fig. 1C,D). Collectively, these results suggest that the recruitment of RECQL4 to DSB sites is a PARylation‐dependent process, which is consistent with previously reported observations [13].

### The amino acids 360–437 region of RECQL4 is sufficient for PARylation‐dependent recruitment to DSB sites

**Fig. 2:**
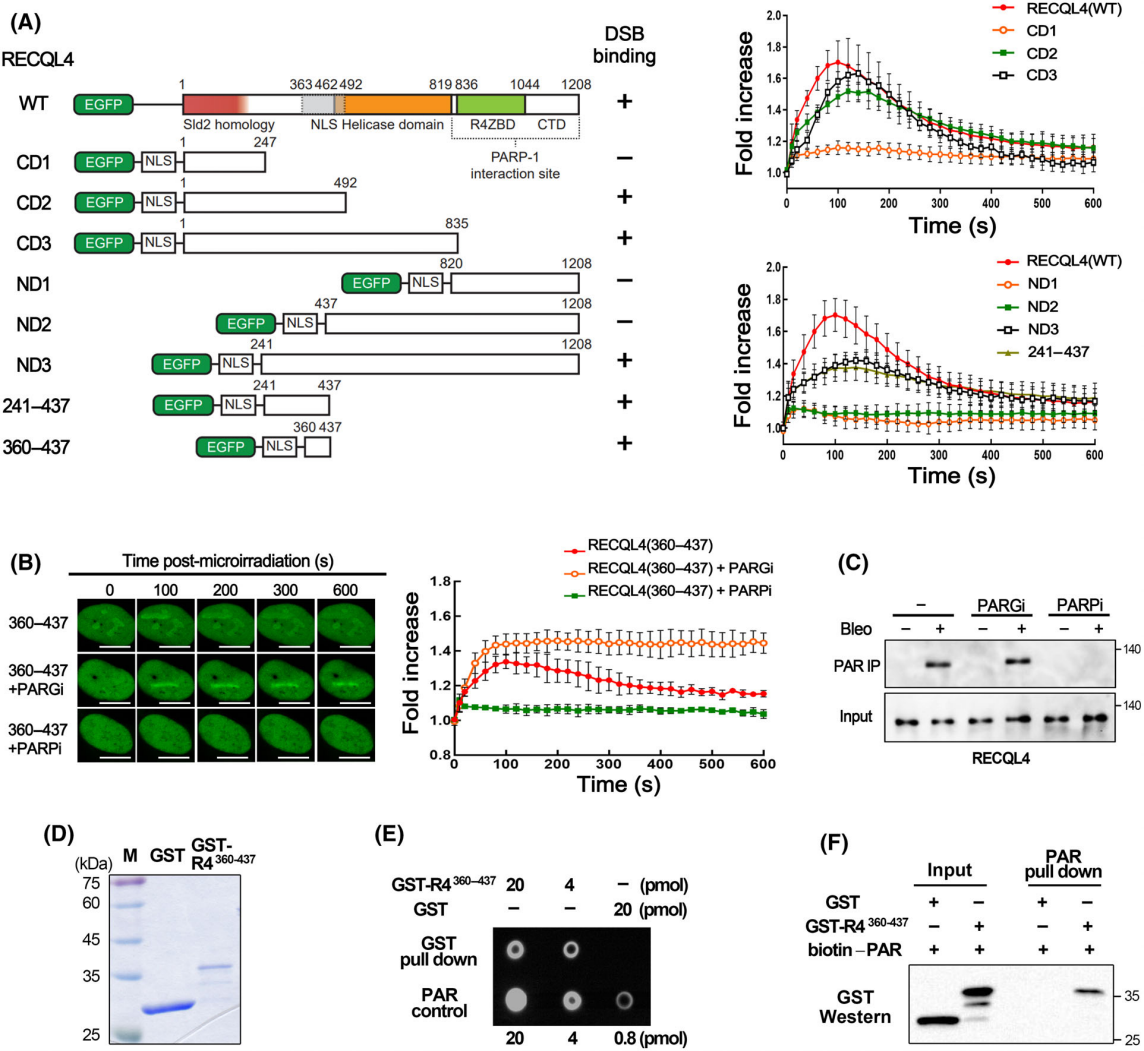
The small region of RECQL4 sufficient for rapid recruitment to DSB sites directly interacts with PAR. (A) Schematic diagram of truncated RECQL4 proteins and their binding to laser‐induced DSB sites and graphs. NLS: the nuclear localization signal sequence of Simian virus 40. Data in graphs are means ± SEM; *n* = 15, from three independent experiments. (B) Recruitment of the region of RECQL4 (aa 360–437) to DSB sites was determined in cells treated with a PARP inhibitor (olaparib) or a PARG inhibitor (PDD000172273). The scale bar represents 10 μm. Data in graphs are means ± SEM; *n* = 15, from three independent experiments. (C) Co‐precipitation of RECQL4 proteins by PAR immunoprecipitation (IP) in cells treated as indicated. Anti‐PAR antibodies were used in IP, and 10% of the extract for IP was used in input lanes. Bleo, bleomycin; (−), untreated. (D) GST and GAT‐RECQL4^360–437^ (GST‐R4^360–437^) proteins were expressed in *Escherichia coli* cells and purified. (E) The PAR polymer (20 pmol) was incubated with the indicated amount of GST or GST‐R4^360–437^ protein, and GST pull‐down assays were carried out. For PAR control, the indicated amount of PAR polymer was directly immobilized onto a nylon membrane. Immunoblotting was carried out with an anti‐PAR antibody. (F) For the PAR pull‐down assay, 30 pmol of biotin‐labeled PAR polymer was incubated with 30 pmol of GST or GST‐R4^360–437^ protein and streptavidin beads. Western blotting was carried out with an anti‐GST antibody. In the input lanes, 25% of input materials were used. (C, E, F) These experiments were conducted more than once and the results of a single experiment are shown.

To clarify the mechanism of PARylation‐dependent recruitment to DSB sites, we mapped the minimal region of RECQL4 required for binding to DSB sites. For this purpose, several EGFP‐fused truncated RECQL4 proteins were generated, and their binding to the microirradiation site was examined using live‐cell imaging. Although the C‐terminal region of RECQL4 was shown to be a PARP‐1 interaction site in a previous study [14], RECQL4‐truncated proteins containing the C‐terminus, such as ND1 and ND2, did not show significant binding to DSBs (Fig. 2A). On the other hand, the truncated RECQL4 protein containing amino acid residues 360–437 was capable of binding to DSB sites (Fig. 2A), and the timing of its binding to and dissociation from DSB sites (Fig. 2B) was similar to that of the full‐length RECQL4 (Fig. 1B). Further, its binding to DSBs was prevented by the inhibition of PARP and maintained by the inhibition of PARG (Fig. 2B), suggesting that its interaction with DSBs also depends on PARylation, as shown in the full‐length RECQL4. Based on these observations, we concluded that the amino acids 360–437 region of RECQL4 contains all the requirements for the rapid, transient, and PARylation‐dependent association of RECQL4 with DSBs.

### The region of RECQL4 sufficient for rapid and transient recruitment to DSB sites directly interacts with PAR

As the small domain of RECQL4 without the PARP‐1 interaction site showed PARylation‐dependent recruitment to DSB sites (Fig. 2A,B), we reasoned that PARylation‐dependent recruitment of RECQL4 is mediated by its interaction with PAR via this domain rather than by its PARylation. As shown in Fig. 2C, immunoprecipitation of PAR in U2OS cells resulted in the co‐precipitation of RECQL4 only in cells treated with bleomycin, a DSB‐inducing reagent. Treatment of a PARG inhibitor did not significantly influence the co‐precipitation of RECQL4, but PARP inhibitor treatment almost completely disrupted the precipitation of RECQL4. These results suggest that RECQL4 stably interacts with PAR in cells containing DSBs. To test whether RECQL4 can interact directly with PAR, we expressed and purified GST fused RECQL4^360–437^ (GST‐RECQL4^360–437^) proteins (Fig. 2D) and examined their physical interactions with the PAR polymer *in vitro*. GST pull‐down and PAR pull‐down resulted in the precipitation of the PAR polymer and GST‐RECQL4^360–437^, respectively, whereas the interaction between the GST protein and PAR polymer was not observed in either experiment (Fig. 2E,F). These results indicate that the domain of RECQL4^360–437^ can interact directly with the PAR polymer.

### RECQL4^360–437^ contains a PBM that is essential for the recruitment of RECQL4 to DSB sites

**Fig. 3:**
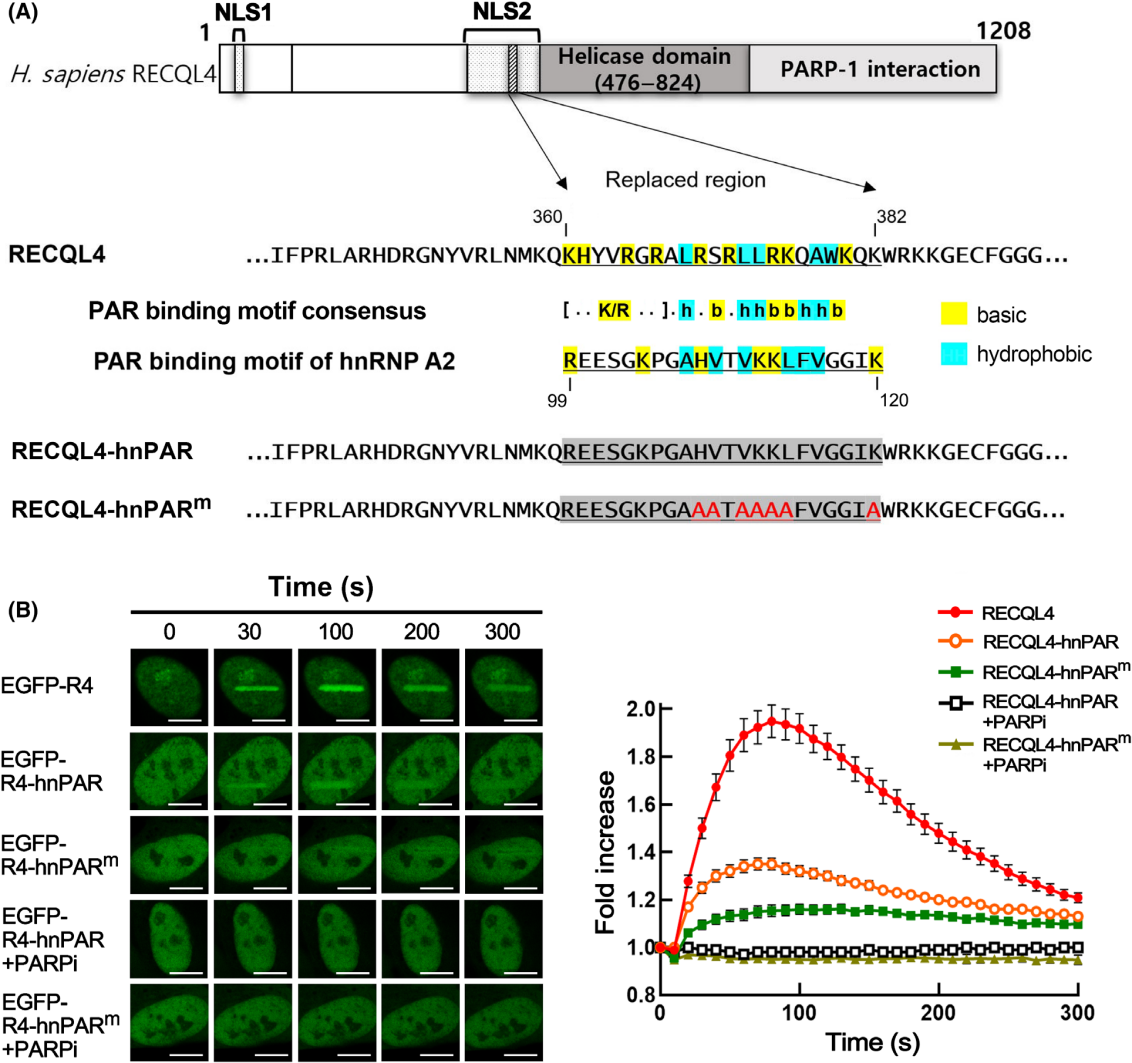
The putative PAR‐binding motif (PBM) in RECQL4 is important for the recruitment of RECQL4 to DSB sites. (A) Schematic diagram of human RECQL4, the amino acid sequence of the putative PBM in RECQL4, and its replacement with wild‐type (RECQL4‐hnPAR) or mutated PBM (RECQL4‐hnPAR^m^) in hnRNPA2. Substituted amino acid positions are indicated as a gray background, and the positions replaced with alanine are indicated in red. Numbers refer to the amino acid positions from the initiation codon. (B) Recruitment of various RECQL4 derivatives to laser‐induced DSB sites in the absence or presence of a PARP inhibitor (olaparib). Representative images (left panel) and graphs (right panel) are shown. R4 stands for RECQL4. The scale bar represents 10 μm. Data in graphs are means ± SEM; *n* ≥ 20, from three independent experiments.

As the region of amino acid residues 360–437 of RECQL4 directly interacts with the PAR polymer *in vitro* (Fig. 2E,F), we carefully examined the amino acid sequence of this region. We found that it contains a putative PBM consisting of positively charged amino acids interspersed with hydrophobic amino acids (Fig. 3A), which shows sequence similarity to the well‐known consensus sequence of PBM [15]. To examine the role of this putative PBM in PARylation‐dependent recruitment of RECQL4 to DSBs, 20 amino acid sequences containing this motif in RECQL4 were replaced with a well‐studied PBM in hnRNPA2 (RECQL4‐hnPAR) or its mutated form (RECQL4‐hnPAR^m^) (Fig. 3A), which was shown to be inactive in PAR binding [16]. As shown in Fig. 3B, the RECQL4 protein with a PBM of hnRNPA2 (EGFP‐R4‐hnPAR) still showed rapid and transient binding to DSB sites in a PARylation‐dependent manner, although the level of protein binding appeared to be relatively low. On the other hand, RECQL4 proteins substituted with a mutant PBM of hnRNPA2 (EGFP‐R4‐hnPAR^m^) did not show rapid binding to DSB sites, although a very low level of binding to DSB sites was still observed (Fig. 3B). These results suggest that the interaction of the PBM in RECQL4 with PAR is important for the rapid and transient recruitment of RECQL4 to DSB sites.

### Interaction of RECQL4 with PAR is important for the DSB response in human cells

**Fig. 4:**
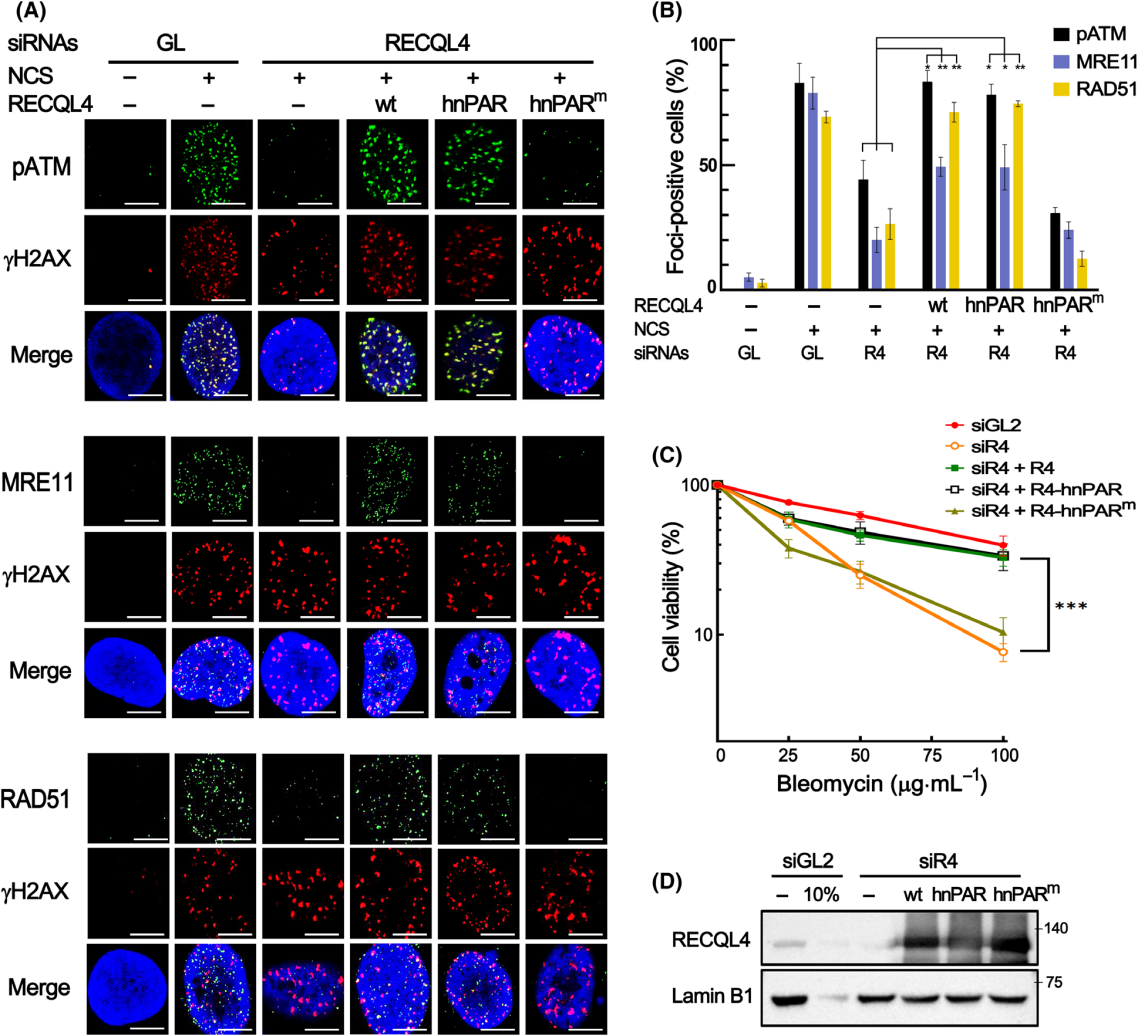
Interaction of RECQL4 with PAR is important for the DSB response in human cells. (A, B) Immunostaining of pATM, MRE11, RAD51, and γH2AX in RECQL4‐depleted (siR4) U2OS cells transfected with wild‐type RECQL4 (R4), RECQL4‐hnPAR (hnPAR), or RECQL4‐hnPAR^m^ (hnPAR^m^). The cells were treated with NCS and incubated in fresh medium for 1 h. Representative images (A) and graphs for foci‐positive cells (B) are shown. Data in graphs are means ± SD; *n* = 3. In each experiment, more than 100 cells were counted. Results of Student's *t* test are shown. **P < 0.01, *P < 0.05. (C) Sensitivity to bleomycin, a DSB‐inducing reagent, of U2OS cells expressing wild‐type RECQL4, RECQL4‐hnPAR, or RECQL4‐hnPAR^m^ proteins in lieu of endogenous RECQL4. WST‐1 assay was carried and data in graphs are means ± SEM; *n* = 3. Result of *t* test between RecQL4‐depleted cells and cells expressing RECQL4‐hnPAR is shown. ***P < 0.001. (D) The western blot showing the depletion of RECQL4 and expression of three forms of RECQL4 proteins.

As the interaction of RECQL4 with PAR appears to be critical for its recruitment to DSB sites, we further examined whether this interaction is also important for the role of RECQL4 in the DSB response. For this purpose, foci of pATM, MRE11, and RAD51, which represent the activation of ATM, stable maintenance of the MRN complex, and HR repair ability, respectively, were observed by immunostaining in cells treated with a DSB‐inducing reagent, NCS. As shown in Fig. 4A,B, NCS‐induced foci of these proteins, which were reduced in RECQL4‐depleted cells, were significantly increased by the expression of wild‐type RECQL4 and RECQL4‐hnPAR. On the other hand, the expression of RECQL4‐hnPAR^m^, containing the inactive mutant PBM of hnRNPA2, did not increase these foci in RECQL4‐depleted cells (Fig. 4A,B). Furthermore, the sensitivity of RECQL4‐depleted cells to a DSB‐inducing reagent, bleomycin, was significantly decreased by the expression of wild‐type RECQL4 as well as RECQL4‐hnPAR, but not by the expression of RECQL4‐hnPAR^m^ (Fig. 4C,D). Taken together, these results suggest that the interaction of RECQL4 with PAR via its PBM is important not only for the recruitment of RECQL4 to DSB sites but also for its roles in the DSB response, such as ATM activation, stable maintenance of the MRN complex, and HR repair.

## Discussion

In this study, we showed that PARylation‐dependent recruitment of RECQL4 to DSB sites through its direct interaction with PAR is critical for proper cellular response to DSBs in human cells. Although RECQL4 was shown to be recruited to DSB sites in a PARylation‐dependent manner [13], this is the first report clearly demonstrating the mechanism and biological significance of PARylation‐dependent recruitment of RECQL4 to DSB sites.

PARylation is a post‐translational modification that plays important roles in the DDR, such as the detection of DNA lesions and the recruitment of DNA repair proteins [17, 18]. The creation of long chains of PAR is catalyzed by PARP in damaged sites, and PARP‐1, a major PARP in human cells, has been shown to play extensive and varied roles in the DNA damage repair pathway [19, 20]. PARylation plays a critical role in recruiting essential PAR‐binding repair proteins, such as XRCC1, to damage sites in the repair of single‐strand breaks and the base excision repair [21, 22, 23]. PARylation was also shown to be important for the recruitment of repair proteins to DSB sites, but its influence on the DSB response appears to be more complicated. DNA DSBs, the most cytotoxic type of DNA damage, can be repaired by various DNA repair pathways, including non‐homologous end joining, alternative end joining, and HR repair, and reportedly, changes in one repair pathway influence the other repair pathways [24, 25]. Given that several proteins involved in these repair pathways, including ATM, MRE11, NBS1, BARD1, BRCA1, BRCA2, and KU70/80, are directly or indirectly controlled by PARP and PARylation [26, 27, 28, 29, 30, 31], it is challenging to determine the biological significance of the PARylation‐dependent control of one repair protein.

In this study, we found that small domain of RECQL4 sufficient for PARylation‐dependent recruitment to DSB sites contained a PBM. The PBM was identified in many nuclear proteins including damage‐related proteins [32], and a typical PBM was characterized by approximately 20 amino acid residues with a cluster of basic amino acids at the N‐terminus as well as an alternating arrangement of hydrophobic and basic amino acids (Fig. 3A) [16]. To examine the cellular role of the PBM in RECQL4 and its interaction with PAR during the DSB response, we substituted this PBM with a well‐characterized PBM of another protein, hnRNPA2. The PBM of hnRNPA2 was identified using a proteomic approach, and alternately arranged hydrophobic and basic amino acids were shown to be essential for its PAR‐binding activity [16]. By examining the biological activity of the RECQL4 protein in which PBM was replaced with wild‐type or mutated PBM of hnRNPA2, we successfully demonstrated that the interaction of RECQL4 with PAR via this PBM is critical not only for DSB binding but also for all known functions of RECQL4 in the DSB response, such as ATM activation, maintenance of MRN stability, and HR repair.

Previously, RECQL4 was shown to interact with PARP‐1, and the C‐terminal domain of RECQL4 was shown to be responsible for this interaction [14]. As RECQL4 physically interacts with PARP‐1, RECQL4 might be recruited to DSB sites via this interaction. However, the C‐terminus of RECQL4, containing PARP‐1 interacting domain alone, did not show any binding to the microirradiation site (Fig. 2A). The RECQL4 protein with the mutated PBM of hnRNP A2 hardly bound to DSBs, and the very low level of residual binding observed with this protein did not occur rapidly (Fig. 3B). Furthermore, recruitment of this protein to DSB sites did not occur in cells treated with olaparib, a PARP inhibitor, resulting in PARP‐1 trapping at DSB sites [33]. This suggests that RECQL4 does not interact with trapped PARP‐1 in DSB sites or that the interaction of RECQL4 with PARP‐1 does not contribute to the recruitment of RECQL4 to DSB sites. Therefore, the interaction of RECQL4 with PAR appears to be solely responsible for its recruitment to DSB sites. Since the C‐terminus of RECQL4 was also shown to be a target of PARP‐1 for PARylation [14], PARylation of RECQL4 recruited to DSB sites may influence the dissociation of RECQL4 from DSB sites or the biological role of RECQL4 in the DSB response.

Although we showed in this study that rapid and transient recruitment of RECQL4 to DSB sites via its interaction with PAR is important for the DSB response in human cells, how RECQL4 and its helicase activity play essential roles in the DSB response during their short stay at DSB sites remains unclear. As RECQL4 has intrinsic DNA helicase activity and DNA annealing activity [34], it has the potential to interact with DNA at damage sites and to participate in the remodeling of DNA ends and in the protein binding to DSBs. RECQL4 was also shown to be required for the recruitment of a deubiquitinase, USP2, which is responsible for stabilizing the MRN complex during the DSB response [12]. Therefore, it is also possible that RECQL4 may influence the ubiquitination control of other repair proteins at DSB sites.

Studies on the PARylation control of the DSB response are important not only to understand genome maintenance mechanisms in cells but also to develop effective cancer therapies. Ever since the specific killing of BRCA1‐ or BRCA2‐deficient tumor cells by PARP inhibition was reported [35, 36], several PARP inhibitors have been successfully developed and used for cancer therapy [37, 38]. Although early studies mostly focused on cancer cells with germ line mutations of BRCA genes, PARP inhibitors have been shown to inhibit the growth of breast cancer cells, regardless of their BRCA status [39], suggesting that chemotherapy using PARP inhibitors is useful for other cancer cells with aberrant DNA damage responses. Therefore, the observations made in this study would be helpful in understanding the PARylation‐dependent control of the DSB response in human cells and developing cancer therapeutics.

## Conflict of interest

The authors declare no conflict of interest.

### Peer review

The peer review history for this article is available at https://www.webofscience.com/api/gateway/wos/peer‐review/10.1002/2211‐5463.13917.

## Author contributions

SS, HK, and J‐KL designed the study. SS, DK, HK, W‐HC and GK performed the experiments. SS, DK, HK, W‐HC, GK, and J‐KL analyzed the data. SS, DK, and J‐KL wrote the manuscript. J‐KL supervised the study.

## Data Availability

The data that support the findings of this study are available from the corresponding author (joonlee@snu.ac.kr) upon reasonable request.
